# The extraction of difficult bile duct stones in a patient with surgically altered anatomy using a novel retrieval basket and a short‐type single‐balloon enteroscopy

**DOI:** 10.1002/jhbp.1427

**Published:** 2024-04-09

**Authors:** Masanari Sekine, Taku Mizutani, Ryo Hashimoto, Goya Sasaki, Azumi Sato, Shu Kojima, Keita Matsumoto, Takeshi Uehara, Takeharu Asano, Hirosato Mashima

**Affiliations:** ^1^ Department of Gastroenterology Jichi Medical University Saitama Medical Center Saitama Japan

## Abstract

Sekine and colleagues report successful stone extraction using a novel retrieval basket and a short‐type single‐balloon enteroscope in a patient with surgically altered anatomy. Its unique shape makes the basket with a 0.018‐inch guidewire the first choice for removal of small bile duct stones in patients with surgically altered anatomy.

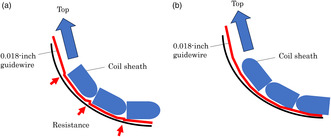

Extraction of common and intrahepatic bile duct stones is generally performed endoscopically. However, stone extraction in patients with surgically altered anatomy (SAA) is sometimes difficult.^1^, ^2^ One reason is that limited devices are available for balloon enteroscopy owing to the length and size of the working channel. A novel retrieval basket with a unique helical shape (VolticCatch V; Olympus Medical Systems, Tokyo, Japan; Figure 1) has been reported to be useful for extracting bile duct stones in a patient with SAA using a colonoscope with a working channel diameter of 3.7 mm.^3^ The previous model of VolticCatch V using a 0.018‐inch guidewire could not pass through a working channel, 3.2 mm in diameter and 152 cm in length, of a short‐type single‐balloon enteroscope (SIF‐H290S; Olympus Medical Systems) in difficult cases, such as making loops, but the improved model could pass through it (Figure 2). We report two cases of successful bile duct stone extraction in patients with SAA using this basket with a 0.018‐inch guidewire and may be the first choice for SAA patients to remove small bile duct stones due to its unique shape.

**FIGURE 1:**
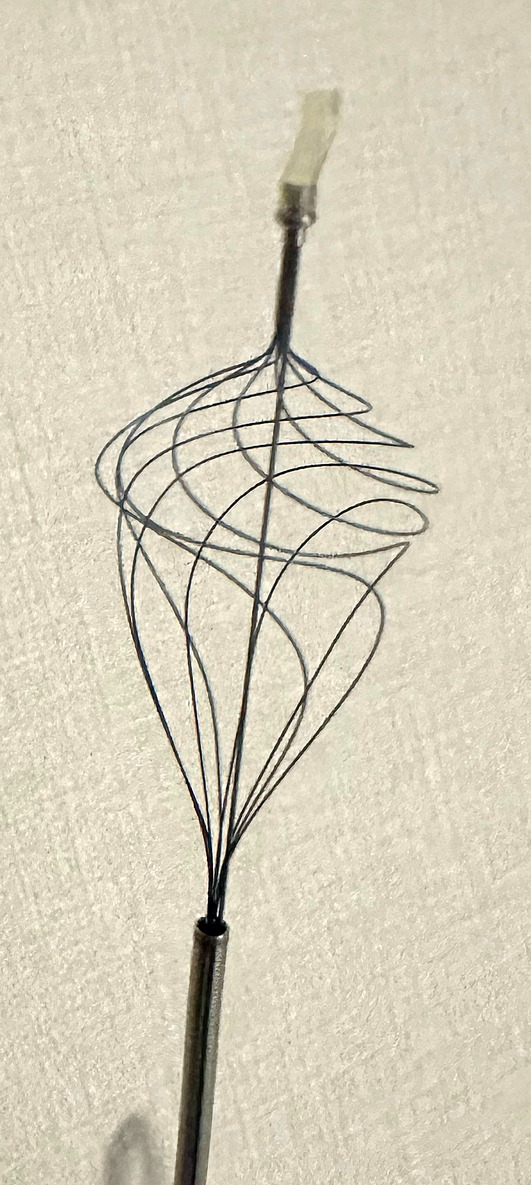
A novel retrieval basket with a unique helical shape (VolticCatch V).

**FIGURE 2:**
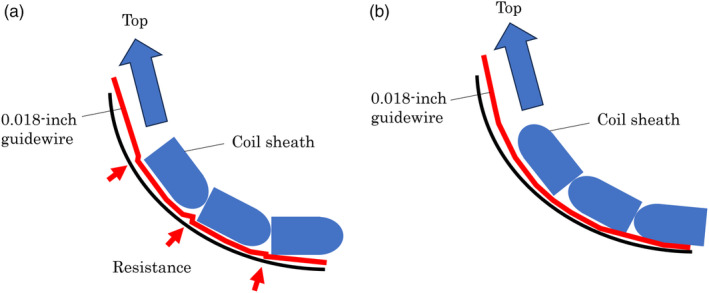
(a) Previous model: a coil sheath was created with the square side at the top and round side at the tail. The resistance to the 0.018‐inch guidewire when inserting the coil sheath was strong in the curve (arrow). (b) Improved model: a coil sheath was created with the round side to the top and square side to the tail. The resistance to the 0.018‐inch guidewire when inserting the coil sheath weakened in the curve.

A 79‐year‐old male was referred for stone extraction. The patient underwent total gastrectomy with Roux‐en‐Y reconstruction for gastric cancer. Endoscopic retrograde cholangiopancreatography (ERCP) was performed using SIF‐H290S. Cholangiography revealed multiple defects. We used a mechanical lithotripter and balloon catheter; however, the stones slipped through the basket and balloon near the papilla. Therefore, a novel basket was used after changing the guidewire from 0.025‐ to 0.018‐inch. Finally, the stones could be removed.

In another case, a 25‐year‐old female underwent choledochojejunostomy for congenital biliary dilatation. ERCP was performed for stone extraction using SIF‐H290S. Cholangiography revealed a defect in the right hepatic duct. We could easily remove intrahepatic bile duct stones using a novel basket with a 0.018‐inch guidewire.

## CONFLICT OF INTEREST STATEMENT

The authors confirm there is no conflict of interest.

## Supporting information


Video S1.
