# Supplementary material for: The extraction of difficult bile duct stones in a patient with surgically altered anatomy using a novel retrieval basket and a short‐type single‐balloon enteroscopy
Source: J Hepatobiliary Pancreat Sci. 2024 Apr 9;31(6):e34–5. doi: 10.1002/jhbp.1427 (PMC11503456; doi:10.1002/jhbp.1427)
Supplement: Supplementary file 1 — Video S1. [file JHBP-31-e34-s001.zip › jhbp1427-sup-0002-Caption.docx]

Video caption

**32-41sec**

The bile duct was cannulated in a retro-flex position.

**42-49sec**

We performed EPLBD without EST.

**50-96sec**

We used a mechanical lithotripter and a balloon catheter, but the stones slipped through the basket and balloon near the papilla.

**97-136sec**

After changing the guidewire from 0.025 inch to 0.018 inch, we used a novel retrieval basket with a unique helical shape (VolticCatch V).

**137-143sec**

Finally, we were able to remove the stones and confirmed no bile duct stones by cholangiography.

**158-166sec**

There was no stenosis at the anastomotic site.

Cholangiography revealed a defect in the right hepatic duct.

**167-176sec**

A novel retrieval basket with a unique helical shape (VolticCatch V) was used with a 0.018-inch guidewire.

**177-184sec**

We were able to remove the intrahepatic bile duct stone.
